# A Volunteer Crossover Feasibility Study to Compare Standard Oxygen Cannula Used at High-Flow to Proprietary High-Flow Humidified Nasal Oxygen Systems

**DOI:** 10.7759/cureus.33738

**Published:** 2023-01-13

**Authors:** Mark Z Johnson, Gary Devine, Rebecca Marshall

**Affiliations:** 1 School of Medicine, University College Dublin, Dublin, IRL; 2 Anesthesia, Fiona Stanley Hospital, Perth, AUS; 3 Anesthesiology, St. Vincent's University Hospital, Dublin, IRL

**Keywords:** anaesthetists, anaesthetics, general critical care, anaesthesia, oxygen inhalation therapy, oxygen

## Abstract

Background and aim: Supplemental oxygen is routinely administered to patients prior to and during induction of general anesthesia and sedation. This increases the fraction of oxygen in the lungs, increases oxygen delivery, and increases the time to oxygen desaturation. Proprietary Transnasal Humidified Rapid-Insufflation Ventilatory Exchange (THRIVE) systems, which deliver warmed and humidified oxygen, have been extensively researched in the perioperative and critical care setting and have been shown to significantly prolong time to desaturation and as a means of ventilatory support. The use of traditional nasal oxygen cannula used at maximum flow rates is currently used in short bursts as it is poorly tolerated. There is however a dearth of data examining the use of this technique. We hypothesized that traditional nasal oxygen cannulae used at maximum flow rates can deliver oxygen as effectively as THRIVE in this setting.

Methods: We designed a crossover volunteer feasibility study. The participants were 10 healthy anesthetists. We compared the two methods of oxygen delivery by measuring transcutaneous oxygen measurement and pharyngeal oxygen concentration. Comfort and noise levels were recorded. The aforementioned parameters were compared between the two groups.

Results: We observed that a standard oxygen cannula used at high flows delivers comparable oxygen delivery and tissue oxygenation performance to proprietary THRIVE systems. However, they are less comfortable and make more noise.

Discussion: To the authors' knowledge this study is the first to study the oxygen delivery of traditional nasal oxygen cannula used at maximum flow rates and make comparisons to the well-studied THRIVE technique. While similar transcutaneous partial pressure of oxygen and pharyngeal gas concentrations were observed with both techniques, the standard cannulae were deemed to be a lot less comfortable than THRIVE and made a lot more noise which likely limit the utility of this technique outside of short bursts.

Conclusion: In this study, a standard nasal oxygen cannula used at high flows achieved similar oxygen delivery to THRIVE at the expense of poor comfort and increased noise.

## Introduction

Supplemental oxygen is routinely administered to patients prior to and during induction of general anesthesia. This practice provides increases the fraction of oxygen in the lungs therefore and providing additional oxygen to the tissues which can prolong the time to oxygen desaturation. In recent years, proprietary high-flow nasal oxygen systems have been extensively researched in the perioperative and critical care setting, they are commonly referred to as Transnasal Humidified Rapid-Insufflation Ventilatory Exchange (THRIVE). They supply warmed, humidified oxygen at up to 70l/min. They have been shown to improve tissue oxygenation as well as significantly delay oxygen desaturation, even in apneic patients, as well as a means of ventilatory support [1-3]. Standard nasal oxygen cannulas are widely used in the healthcare setting since their introduction in the 1940s and have remained largely unchanged to the present day. Their use at lower flows, at up to 10 L/min, has been studied and has demonstrated a modest increase in time to oxygen desaturation when used at the time of induction of anesthesia [4,5]. The use of supplemental nasal oxygen during tracheal intubation is supported by societal guidelines [6]. Among critically unwell patients, THRIVE has been shown to perform similarly to bag-mask ventilation in preventing oxygen desaturation during tracheal intubation [7-9]. In prior studies, THRIVE has been shown to perform similarly to standard face mask pre-oxygenation [10,11]. We hypothesized that standard nasal cannula could deliver oxygen as effectively as THRIVE in this setting, with the benefits of being quick to set up, widely available, and for a small fraction of the cost. In this study, we aimed to compare oxygen delivery between standard nasal oxygen cannula delivering dry, non-warmed oxygen at maximum available flow rates with the “Optiflow” THRIVE system (Fisher and Paykel, Auckland, New Zealand) by measuring the transcutaneous partial pressure of oxygen and pharyngeal gas concentrations.

## Materials and methods

The South Metropolitan Health Service Ethics Board in Perth, Western Australia, approved this study (PRN: RGS0000001177). Our study consisted of two phases. Phase one involved establishing the maximum available flow rates achievable from commonly available gas outlets. This was performed using a gas flow sensor (VT900A Gas Flow Analyser Ventilator Tester, Fluke Biomedical, Washington, DC, USA.). Oxygen taps were turned clockwise until they stopped turning. The flow meter was connected using standard oxygen tubing.

Phase two was a volunteer study comparing standard nasal oxygen cannula at maximum flows and THRIVE. We aimed to examine if the standard cannula were non-inferior to THRIVE. We recruited 10 healthy volunteer anesthetists aged 18 to 65, of ASA class 1, who were non-obese and who gave informed written consent. Recruitment was stopped prematurely with 10 of the planned 20 participants recruited with the declaration of the SARS-CoV-2 pandemic in 2020. Participants lay on a theatre table at 45°. Their nasal passage of choice was tropicalized with co-phenylalanine spray. A gas sampling catheter was advanced through the nose until its tip was visualized in the low oropharynx. This method of gas sampling was employed as it measured similar expired CO2 concentrations as tidal breathing without oxygen supplementation, unlike more distal sampling points such as the nasopharynx. A transcutaneous oxygen partial pressure (TCpO2) sensor (TCM4 CombiM; Radiometer Medical ApS, Brønshøj, Denmark) was attached to the participant in the infraclavicular fossa. TcpO2 monitoring was used in preference to oxygen saturation (SpO2) as SpO2, although used widely in clinical practice, would not show any differences between treatments in spontaneously breathing participants receiving supplemental oxygen. The monitor was allowed to reach a steady state. Participants were randomized to receive either standard nasal oxygen prongs at maximum flow via the “Christmas tree” oxygen outlet on the anesthetic machine (Zeus, Draeger, Lübeck, Germany), or 100% oxygen via Optiflow at 60l/min. Randomization was done via coin toss. All participants received both treatments. There was a 10-minute washout time and TcpO_2_ was confirmed to have returned to steady state between treatments. Pharyngeal gases were sampled via the gas sampling line of the Zeus machine attached to the pharyngeal catheter. Participants were instructed to breathe only through their nose with their mouth closed. Both treatments had a duration of 3 minutes. The three-minute time frame was based on conventional preoxygenation timescale as well as an observed plateauing of TcpO_2_ in preliminary work. At the end of each therapy, participants were asked to score the comfort rating by marking an X along a 100mm line marked “very comfortable” at the left and “very uncomfortable” on the right after each therapy. Sound intensity was measured using a phone-based application “Sound Meter - Decibel” (Melonsoft, Bangkok, Thailand). All data were analyzed using GraphPad Prism Statistics. Continuous data was compared using paired Student’s t-test; p < 0.05 was considered statistically significant.

## Results

For phase 1, we measured maximum flows from numerous anesthetic machines and commonly used “Christmas tree” outlets. The results are described in Table 1.

**Table 1:** The maximum flow rates available from commonly available “Christmas tree” oxygen outlets.

Outlet	Maximum Flow (l/min)
Drager Zeus	61
Drager Fabius	15
Datex-Ohmeda Aisys CS2	60
Datex-Ohmeda Aespire S/5	60
Wall Thorpe tube	21

**Figure 1:**
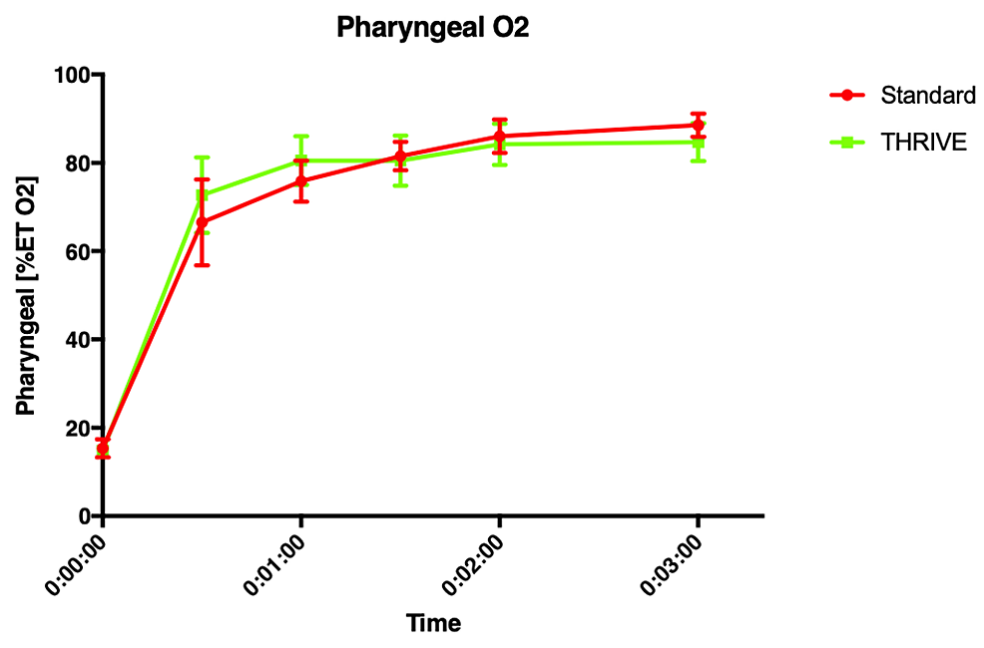
Mean (SD) end-tidal pharyngeal oxygen percentage vs time.

**Figure 2:**
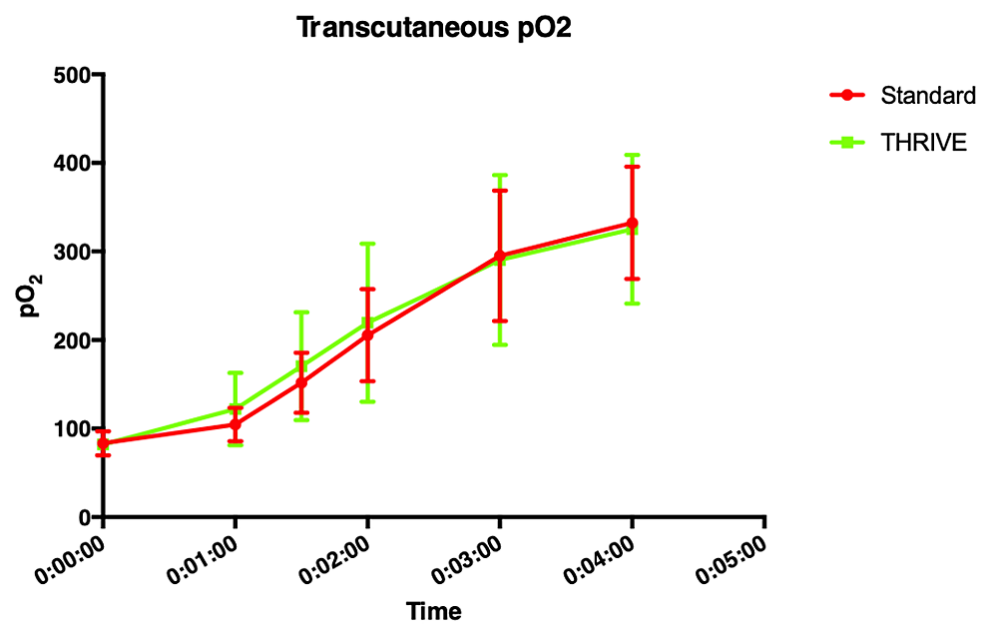
Mean (SD) transcutaneous oxygen partial pressure vs. time.

**Figure 3:**
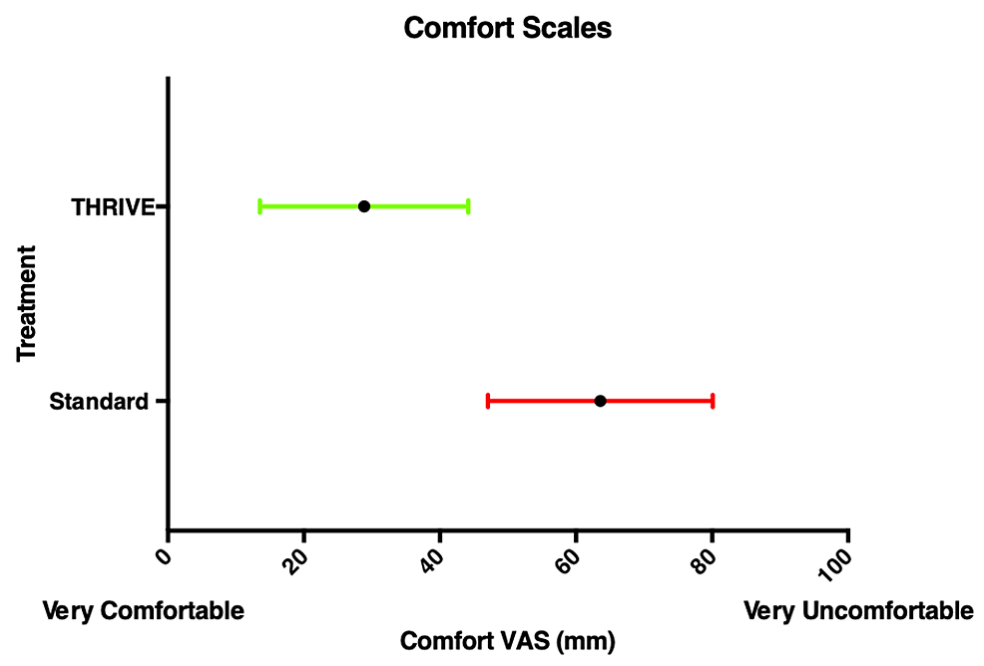
Mean (SD) comfort scores reported by study participants.

For phase 2, we recruited 10 volunteers for the study, one of whom did not tolerate the pharyngeal catheter so only TcpO_2_ data was available for this subject. The participants included three females and seven males with median (range) ages 35 (28-45) years and BMI 23.0 (21-26) kg/m^2^. Peak TcpO_2_ lagged behind the cessation of treatment, generally by 20 to 60 seconds until peak value was recorded. This is consistent with prior studies using the same device [10]. There were no significant differences between the two methods in terms of pharyngeal expired oxygen concentration (Figure 1), with mean peak expired oxygen concentration of (mean [SD]) 88.5% (2.6%) in the standard nasal cannula group compared with 84.7% (4.3%) in the THRIVE group (p=0.09) and peak TcpO2 of 332kPa (63kPa) and 325kPa (84kPa) (p=0.86) respectively (Figure 2). The THRIVE system was significantly more comfortable than the standard nasal cannula, visual comfort scale 29mm (15mm) vs 64mm (15mm) (p<0.0001) (Figure 3) and made less noise 75dB vs 88dB, respectively.

## Discussion

Numerous studies in the perioperative and critical care settings have shown the potential uses of high-flow nasal oxygen as a technique for preoxygenation, an alternative to bag-mask ventilation and ventilatory support [7-11]. This study demonstrated that standard oxygen cannula used at high flows have similar oxygen delivery performance to the THRIVE system, at the expense of poorer patient comfort and more noise. This presents an option for clinicians to get the well-described benefits of THRIVE for numerous purposes including preoxygenation, difficult airway management, induction of patients with high metabolic rates, and use with procedural sedation for short-term use.

Benefits of this system include being inexpensive, almost universally available, and fast to set up without expertise, clutter, water, or electricity. There are of course drawbacks including decreased comfort, increased noise levels, and mucosal drying. While these drawbacks likely rule out this method for prolonged periods, especially in fully awake patients, there are benefits to using a standard cannula in the setting of anesthetic induction, emergency airway management, and under sedation. The simplicity and rapidity of its use lend themselves well to employing this technique for urgent theatre cases, and emergency and critical care settings. The presence of the cannula does not significantly inhibit the formation of a seal with a standard face mask to the same extent as the larger diameter “Optiflow” tubing.

There are numerous limitations to our study. They include a small sample of healthy, slim, cooperative, well-informed, and fully conscious subjects. There is potential for bias in this volunteer cohort as anesthetists would expect THRIVE to be more comfortable as well as the data to be collected in an unblinded fashion.

Further studies using this technique in real-world conditions are warranted. A study using oxygen saturation as the primary endpoint would improve clinical correlation and translation of this technique. It is plausible that lower flow rates could potentially infer similar benefits with better comfort scores warranting performing an optimal dose-finding study using this experimental model.

## Conclusions

Many commonly found oxygen outlets are capable of supplying high flows. In our study, a standard nasal oxygen cannula used at high flows performed similarly to proprietary high-flow systems in terms of oxygen delivery. However, this technique is less comfortable and makes more noise than proprietary systems. There may be a role for this technique in patients receiving sedation or anesthesia as well as potential short-term and urgent applications. Further investigation of this technique in a real-world setting is warranted.
